# Who should get the vaccine first? A glimpse at COVID-19 vaccination prioritization strategies

**DOI:** 10.17179/excli2021-3570

**Published:** 2021-03-15

**Authors:** Mehrdad Askarian, Amirhossein Erfani, Mohammad Hossein Taghrir

**Affiliations:** 1 Department of Community Medicine, School of Medicine, Shiraz University of Medical Sciences, Shiraz, Iran; Health Behavior Science Research Center, Shiraz University of Medical Sciences, Shiraz, Iran; 2 Thoracic and Vascular Surgery Research Center, Shiraz University of Medical Sciences, Shiraz, Iran; 3 Student Research Committee, Shiraz University of Medical Sciences, Shiraz, Iran; 4 Department of Community Medicine, School of Medicine, Shiraz University of Medical Sciences, Shiraz, Iran

## ⁯⁯⁯

***Dear Editor,***

Despite the enormous efforts, novel coronavirus disease 2019 (COVID-19) continues to spread worldwide, infecting individuals and causing health and economic burdens. Preventive measures such as wearing a mask, practicing social distancing, utilizing hand sanitizers, isolating patients and quarantine have been somewhat effective in flattening the pandemic curve, yet, they are not the ultimate solution. 

Vaccination as a safe and effective method of stopping the COVID-19 pandemic was initiated in many countries worldwide to reduce transmission, morbidity, and mortality. However, the lack of sufficient supply and distribution to meet the demands in the early stages of vaccine production makes the vaccination process a challenge for communities. As a result, vaccine administration should be done over time with prioritization as the vaccine production and distribution capacity increases. Nevertheless, the question that remains is who to be vaccinated first.

To answer this question, we take a scoping glimpse at the literature. We found no governmental instruction for COVID vaccine prioritiziation in PubMed, Embase and Web of Science. Since the governmental documents usually are addressed in the gray literature, we searched via Google Scholar and Google search engine using the following terms: (“COVID-19” OR “COVID19” OR “SARS-Cov-2”) AND (“Vaccine” OR “Vaccination”) AND (“Priority” OR “Prioritisation”). Finally, We included and tabulated seven instructions implemented or recommended by global health organizations and countries.

So far, several vaccination strategies have been designed by health ministries and organizations, most of which prioritizing front-line healthcare workers, the residence of long-term care facilities, elderlies, and those with underlying medical conditions. Moreover, the World Health Organization (WHO) suggested that the specific age cut-off needs to be decided by locals. Some non-healthcare workers who are called front-line workers or essential workers are also found in the high priority groups, encompassing fire fighters, police officers, corrections officers, food and agricultural workers, Postal Service workers, manufacturing workers, grocery store workers, public transit workers, those who work in the educational sector (teachers, support staff, and daycare workers), people who work in transportation and logistics, food service, housing construction and finance, information technology, communications, energy, law, media, public safety, and public health. Some particular populations, such as indigenous communities were spotlighted, which could be exemplary for other countries and districts to pay special attention to these groups. More details are tabulated in Table 1(Tab. 1) (References in Table 1: Australian Government, Department of Health, 2021[1]; Brent, 2021[2]; CDC, 2021[3]; Government of Canada, 2020[4]; Knight 2020[5]; Public Health England, 2021[6]; WHO, 2020[7]).

It should be mentioned that these strategies may be revised as the vaccination program proceeds if individuals in a targeted group such as those with underlying medical conditions show adverse effects of the vaccine as not all medical conditions have been put in clinical trials in the early phases of vaccine production. Moreover, children under 18 years of age and pregnant women have not been listed as the targeted groups in vaccination strategies due to a lack of information on vaccine administrations' safety and efficacy. Further studies, including these two groups are necessary as these groups can increase the risk of transmission and are vulnerable to contracting the severe form of the disease. 

Early vaccine administration strategies have been designed with the goals of protecting those who are more vulnerable to severe illness (reducing the years of life lost and improving the quality of life), reducing the transmission of COVID-19, protecting healthcare capacities, and protecting the critical infrastructures. Thus, in this manner, these targeted groups must be identified in each region before the initiation of vaccination in order to improve the efficacy of the vaccination process. 

## Conflict of interest

The authors declare no conflict of interest.

## Figures and Tables

**Table 1:**
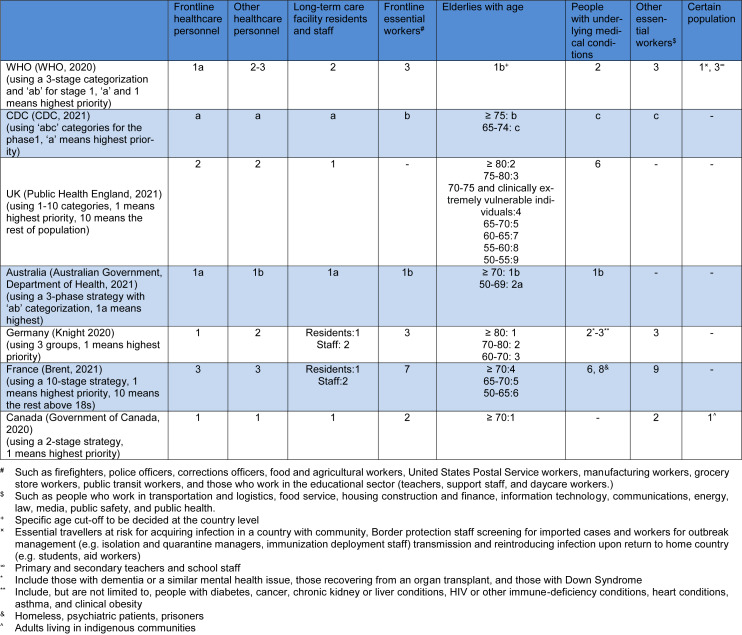
An overview of vaccination strategies in the early phases of vaccine production
